# DefaceQA - automated quality assessment of brain MRI defacing software

**DOI:** 10.1186/s12880-026-02207-4

**Published:** 2026-02-07

**Authors:** Maryam Khodaei Dolouei, Sina Sadeghi, Toralf Kirsten

**Affiliations:** 1 Department for Medical Data Science, Leipzig University Medical Center, Leipzig, Germany; 2 Institute for Medical Informatics, Statistics and Epidemiology, Leipzig University, Leipzig, Germany; 3 Faculty Applied Computer and Bio Sciences, Mittweida University of Applied Sciences, Mittweida, Germany

**Keywords:** Patient privacy, Defacing software, Brain MRI, Machine learning

## Abstract

Defacing of brain magnetic resonance imaging (MRI) scans by removing identifiable facial features is essential for protecting patient privacy, yet assessing defacing quality remains challenging. While deep learning methods offer solutions, they require large labeled datasets, limiting their practical applicability. This study presents *DefaceQA*, a machine learning (ML) approach for automated defacing quality assessment using quantitative image features. A dataset of 200 MRI scans from the Leukodystrophy Registry at the Leipzig University Medical Center was processed using four defacing algorithms: *PyDeface*, *QuickShear*, *FSL-Deface*, and *MRI-Deface*. Image features extracted from original and defaced scans were used to classify defacing efficacy. The ML classifiers achieved an AUROC of 0.84 and an accuracy of 0.85 under a lenient criterion for successful/unsuccessful defacing, with the Feature Similarity Index Measure (FSIM) emerging as a key predictor. The findings demonstrate ML’s potential for defacing evaluation while highlighting challenges related to dataset limitations and generalizability.

## Introduction

Collaborative data-sharing initiatives are imperative in the field of neuroscience, especially in advancing research on rare neurological diseases, where limited case availability hinders progress [1, 2]. These collaborations facilitate advancements in diagnostics, treatments, and the understanding of cognitive and behavioral disorders. However, they also pose significant ethical challenges regarding patient privacy [3]. Magnetic Resonance Imaging (MRI) scans of the brains (heads), widely employed in neuroscience research, include facial features that create a substantial risk of re-identification when rendered in three-dimensional (3D) images. The rapid advancements in facial recognition technology, combined with increasing image resolution, have rendered traditional anonymization techniques, involving removal of sensitive metadata, inadequate [4, 5]. Research findings indicate that reconstructed facial images derived from MRI scans can exhibit a high degree of similarity to full-face photographs [6]. Furthermore, facial recognition software has demonstrated a capacity to successfully match 3D MRI renders to corresponding photographs in 83% of cases [7].

**Fig. 1:**
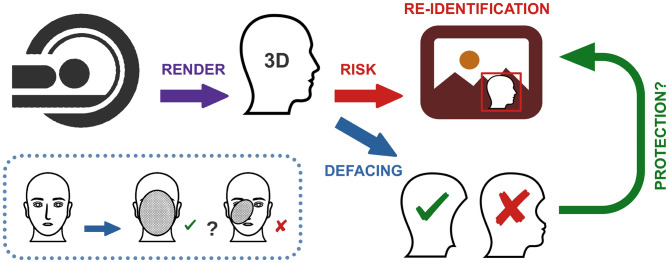
Schematic overview of the defacing process and its limitations. Facial features reconstructed from MRI scans present a significant re-identification risk. Defacing algorithms mitigate this risk by removing identifiable facial features to protect patient confidentiality. Imperfections in the defacing process, however, may leave residual facial features, underscoring the critical need for robust QA tools

Ethical regulations, such as the Health Insurance Portability and Accountability Act (HIPAA) [8] and the General Data Protection Regulation (GDPR) [9], mandate the protection of personal data by requiring the removal of identifiable features from medical images [10, 11]. For this purpose, defacing algorithms were developed to eliminate facial features from MRI scans. However, these algorithms are not without limitations, since they may leave residual facial features, as illustrated in Fig. 1, or inadvertently damage brain tissue, thereby compromising both patient privacy and the scientific utility of the data [12]. The quality assessment (QA) of defaced brain MRI scans remains largely a manual process [12–14], even in large-scale neuroscience initiatives like the Human Connectome Project [15]. While existing research primarily evaluates the impact of defacing on downstream neuroimaging analyses, such as brain segmentation [16], systematic investigations into the efficacy of defacing for privacy preservation remain limited. Recent advances in automated QA methods leveraging deep learning (DL) have demonstrated promising results [17]. However, their reliance on large, manually labeled datasets constrains their scalability and adaptability to emerging defacing techniques. This highlights the need for more data-efficient and generalizable approaches to ensure robust and scalable defacing QA.

In this study, we present the DefaceQA, a novel machine learning (ML) approach for automating the assessment of defacing efficacy in brain MRI scans. This approach utilizes extracted features to quantify the effects of defacing, thereby enabling supervised learning models to evaluate defacing quality with reduced reliance on large-scale manual labeling. The model compares original and defaced MRI scans, determining the adequacy of defacing by referencing manually established ground truth labels. In contrast to DL methods, which typically require extensive labeled datasets, DefaceQA is more resource-efficient and adaptable, making it well-suited for assessing a diverse range of defacing algorithms. This study has the potential to advance scalable, efficient solutions for automated QA in defacing processes, contributing to the evolution of defacing evaluation methodologies in neuroimaging research.

## Methods

**Fig. 2:**
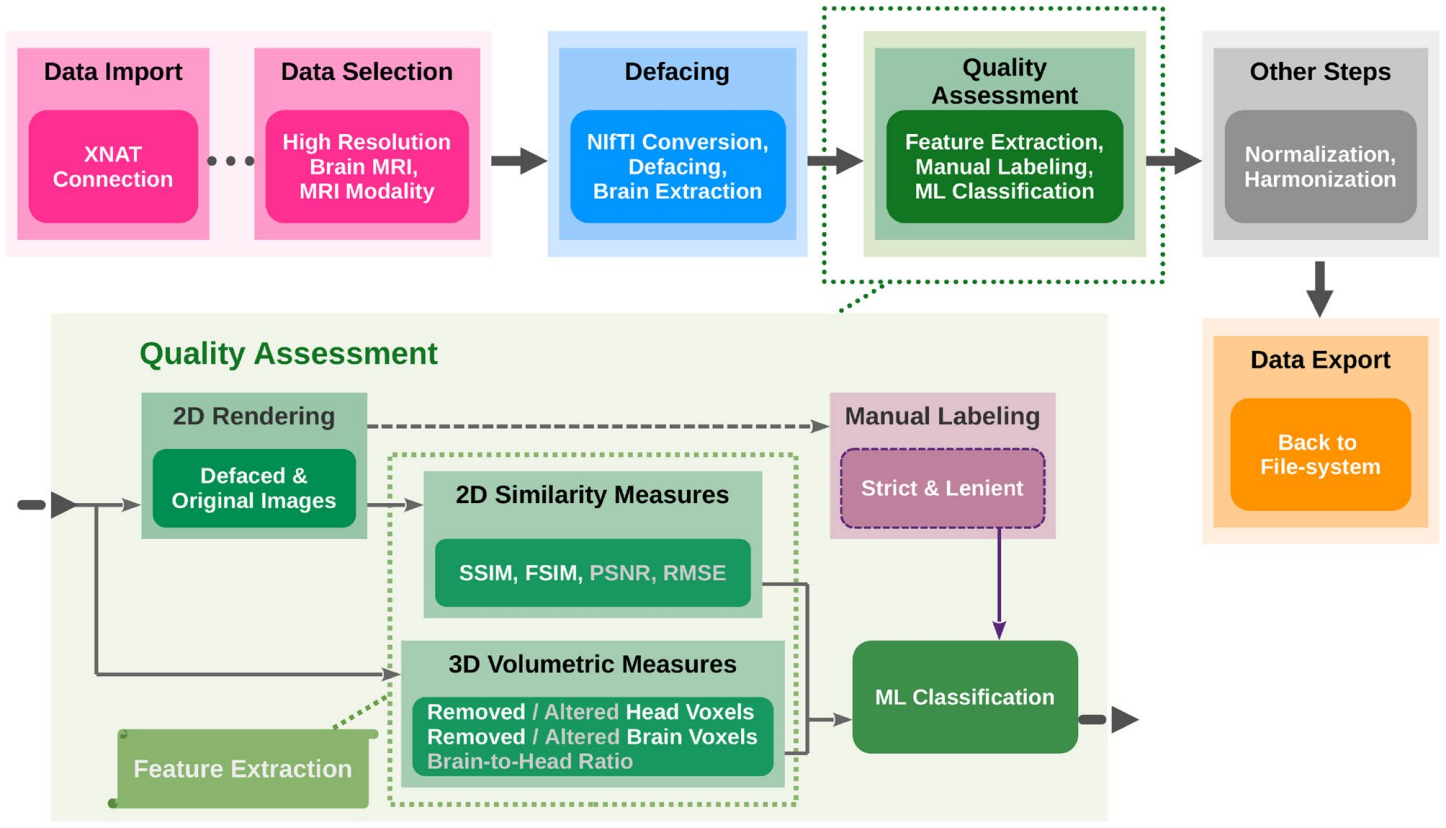
Workflow for data collection, preparation, defacing, and evaluation. The pipeline begins with data import and filtering, followed by defacing and the brain extraction. QA incorporates feature extraction, manual labeling, and ML classification to assess the defacing quality. Features not utilized in the primary model are indicated by a gray color scheme. The outputs may be normalized and harmonized prior to export for downstream analysis

The methodological framework employed by DefaceQA to evaluate defacing success through quantitative metrics and automated ML classification is outlined in Fig. 2. The workflow delineates processes, including data collection, preparation, defacing, and ML modeling pipeline for defacing QA. Key attributes of defaced MRI scans were systematically extracted based on their relevance to the degree of de-identification. The success of defacing was manually assessed using a custom-designed graphical user interface (GUI), thereby creating a labeled dataset. This dataset was then preprocessed and utilized to train multiple ML models, whose performance was rigorously evaluated to determine their predictive accuracy.

### Data import

The MRI data utilized in this study were obtained from the Leukodystrophy Registry of the Clinic and Polyclinic for Neurology at the Leipzig University Medical Center. The MRIs were archived within the open-source imaging-data-management-platform XNAT [18], and were retrieved using an XNAT Python client [19]. The Python client facilitated the direct reading of the Digital Imaging and Communications in Medicine (DICOM) properties of the MRI scans from the server and their subsequent storage. The generated file provided an initial overview of the available MRI scans, guiding the selection of MRIs for inclusion in the experiment. This approach ensured that only relevant data were processed, thereby optimizing computational efficiency. A total of 273 high-resolution 3D head MRI scans were selected, comprising 171 FLAIR and 102 T1-weighted images.

### Data preparation and defacing algorithms

In this study, four widely used defacing algorithms: PyDeface, QuickShear, FSL-Deface, and MRI-Deface were employed to enable methodological diversity in facial feature removal [20–23]. These algorithms were selected based on their distinct approaches and widespread adoption in neuroimaging research, ensuring the robustness of ML classifiers across varying defacing methods [12]. Their use as standard tools within the research community highlights their reliability and acceptance for de-identification in MRI data. As these defacing algorithms operate exclusively on the Neuroimaging Informatics Technology Initiative (NIfTI) file format, the downloaded DICOM scans were converted to NIfTI. This conversion allowed for compatibility with defacing tools while reducing metadata content to increase data anonymity. Duplicate scans were removed during the preprocessing step, resulting in a final dataset of 248 distinct scans.

The four defacing algorithms were then applied to the NIfTI files. Defacing, typically performed via terminal commands, was automated using Python scripts for efficiency and reproducibility. Each defaced file was annotated with a suffix indicating the applied method (e.g., pydeface, quickshear, fsldeface, or mrideface). Notably, QuickShear required a pre-generated brain mask, which was created using FSL’s Brain Extraction Tool (BET) [24, 25]. Following additional exclusion criteria, as detailed in the next section (manual ground truth labeling), 200 out of 248 scans were retained for the final analysis, comprising 139 FLAIR and 61 T1-weighted scans.

### Manual ground truth labeling for supervised learning

To provide ground truth labels for supervised ML model training, all defaced MRI scans were manually reviewed and labeled as either successfully defaced (0) or insufficiently defaced (1). To further facilitate this process and enable the extraction of similarity measures for ML classification, MRI scans were 3D-rendered using the Python libraries PyVista and NiBabel [26, 27]. The resulting facial volumes were converted into two-dimensional (2D) images from five standardized perspectives: frontal, left and right profiles, and left and right 45^∘^ angles. The extracted images were then scaled to the volume boundaries to maintain consistency in the evaluation.

A customized GUI was developed to streamline the classification process. The GUI displays the defaced images alongside their original counterparts for side-by-side comparison, enabling reviewers to assess the efficacy of the defacing. This comparative approach is especially crucial in complex cases, such as distinguishing between internal and surface eye structures or identifying instances of partial feature removal. The defacing efficacy has been independently assessed by three evaluators, two primary raters who reviewed all scans, and a third rater who adjudicated discrepant cases, and the remaining disagreements were resolved by consensus.

Two classification criteria were applied: strict and lenient. The strict criterion required the complete removal of all facial features, including the mouth, nose, eyes and associated superficial tissues, along with the majority of the cheek and jaw regions. In contrast, the lenient criterion permitted minimal residual traces of a single facial feature (e.g., partially visible eyelids) while ensuring the substantial removal of overall facial structures. This dual-criterion approach facilitates a nuanced assessment of defacing efficacy, with the lenient criterion aligning more closely with methodologies from previous studies while maintaining a comparatively higher level of stringency [12].

**Fig. 3:**
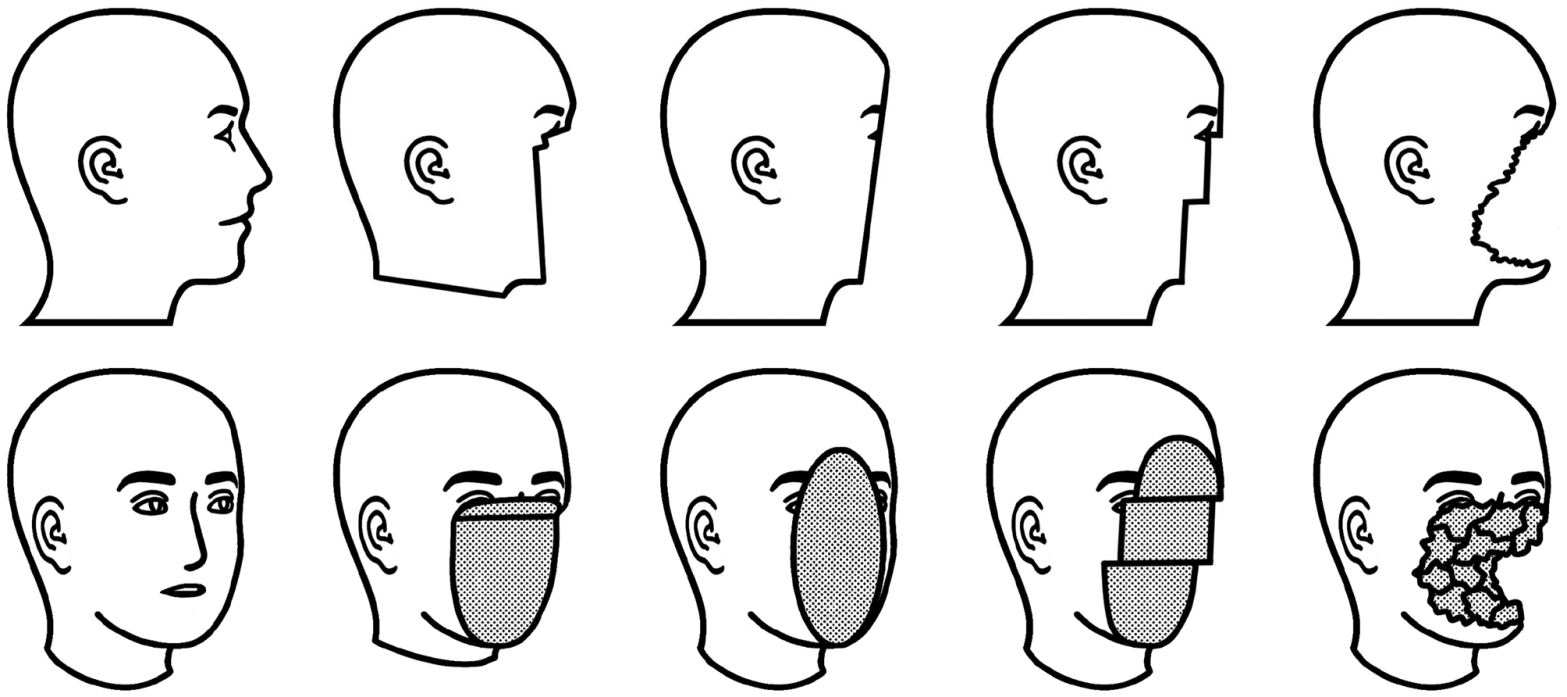
Schematic illustration of sample outcomes from four defacing algorithms, displayed from left to right: the original (non-defaced) image, PyDeface, QuickShear, FSL-Deface, and MRI-Deface, adhering to the lenient defacing criteria. The most frequently retained facial structures were those resembling eyelids, although residual features were also occasionally observed in the lower lip and nasal tip regions under lenient assessment conditions

The GUI also enabled reviewers to exclude scans deemed unsuitable for evaluation, such as pre-defaced originals, incomplete head scans, and those affected by rendering artifacts. This refinement process resulted in a final dataset of 800 defaced scans derived from 200 original MRI scans. Figure 3 presents a schematic illustration of potential defacing outcomes produced by the four defacing algorithms assessed in this study. The figure underscores the complexity of this classification task, even for human raters, due to the gradual transitions between categories and the presence of edge cases, contrasting with classification tasks that involve clearly distinct classes, such as differentiating between cats and dogs.

### Feature extraction - quantitative measures of defacing impact

To systematically assess the impact of defacing, quantitative features were extracted from both 3D MRI volume and 2D surface representations. These features can be categorized into two distinct groups: volumetric measures and similarity metrics. The primary objective was to identify features capable of differentiating between adequately and inadequately defaced scans, with their relevance assessed during the training and testing of ML models. Additionally, the specific defacing method applied to each scan was incorporated as a feature to account for method-specific variations. To mitigate artifacts in both 2D and 3D images, a threshold of 0.03 was applied to the normalized data, effectively removing low-intensity voxels (or pixels). This preprocessing step enhanced the accuracy of the head mask by minimizing background noise and improving segmentation fidelity.

#### Volumetric measures

A volumetric analysis was performed to quantify alterations in 3D MRI volumes resulting from defacing, including the following measures:

- Impacted Voxels: The absolute difference between the original and defaced arrays was computed, yielding the total number of altered voxels. To standardize this metric across varying file dimensions and head sizes, the percentage of impacted voxels relative to total head voxels was calculated.
- Removed Voxels: The percentage of completely removed voxels was calculated by comparing zero values in the original and defaced arrays.
- Brain Voxel Impact: To assess whether defacing affected brain tissue, the overlap between altered voxels and the corresponding brain mask was measured, inspired by Alfaro-Almagro et al. [22]. The percentage of altered and completely removed brain voxels relative to total brain voxels was computed.
- Brain-to-Head Ratio (BHR): The ratio of brain voxels to total head voxels was calculated to detect anomalies in brain mask size.

#### Similarity metrics

Similarity metrics were computed from the 2D images of the defaced and original MRIs to assess the degree of visual similarity between the defaced and original images. To this end, the following measures were calculated using Python’s Image Similarity Measures library [28]: Root Mean Squared Error (RMSE), Peak Signal-to-Noise Ratio (PSNR), Structural Similarity Index Measure (SSIM), and Feature Similarity Index Measure (FSIM) [29].

### Machine learning modeling and evaluation

The final dataset for the analysis consisted of 200 MRI scans, each processed using four defacing methods, resulting in a total of 800 entries. After excluding non-informative metadata and manually assigned classification properties, 11 key quantitative features were retained, along with a binary classification label denoting the success or failure of the defacing process. Under both strict and lenient classification criteria, the dataset remained relatively balanced, with a distribution of approximately 40/60%. The lenient criterion tended to classify defacing outcomes as successful more frequently, whereas the strict criterion more identified a higher proportion of outputs as inadequate.

Four ML models – Logistic Regression (LR), Multi-layer Perceptron (MLP), Random Forest (RF), and Extreme Gradient Boosting (XGB) – were utilized for binary classification of defacing success. To ensure robust and unbiased performance evaluation while preventing data leakage during model development, we implemented a nested stratified group k-fold cross-validation framework [30]. This nested design provides a rigorous estimate of generalization performance by fully isolating the test data from all stages of model selection and optimization [31, 32].

In this framework, an outer loop partitioned the dataset into independent training and test folds, while an inner loop was used exclusively within the training folds for feature selection and hyperparameter tuning. The optimal model identified in the inner loop was then retrained on the full training subset and evaluated on the corresponding outer test fold. This process was repeated across all outer folds to ensure that each data point contributed exactly once for testing.

The group constraint enforced subject-level separation, assigning all scans from a given participant to the same fold and thereby eliminating non-independence leakage, while the stratified component preserved class distribution across folds. Final performance metrics, including accuracy, sensitivity, specificity, precision, and the area under the receiver operating characteristic curve (AUROC), were averaged across all outer folds to provide a reliable summary of classification performance.

Prior to finalizing the ML modeling, an iterative feature selection process was performed to enhance model performance and computational efficiency by minimizing redundancy. Tree-based ensemble models were employed to calculate permutation feature importance, and importance scores were averaged across outer cross-validation folds. Additionally, hierarchical clustering of Spearman rank-order correlations identified highly correlated features, which were systematically removed if they exhibited low importance or strong interdependencies. The models were retrained with the refined feature set, and this process was repeated until an optimal subset was obtained, balancing predictive accuracy and feature redundancy. To assess the generalizability of the models across different defacing methods, their performance was assessed both with and without explicit information about the applied defacing algorithm. This analysis aimed to determine whether an algorithm-independent model could achieve reliable predictions, thereby ensuring broader applicability across a range of defacing approaches.

## Results

The results include an analysis of the dataset’s demographic characteristics and distribution, followed by a performance evaluation of defacing algorithms using human-annotated ground truth labels. Additionally, the study assessed the classification performance of ML models in differentiating adequately from inadequately defaced MRI scans. The findings provide valuable insights into the efficacy of defacing methods and the capacity of ML models for automated defacing QA in MRI defacing.

### Cohort demographics and data characteristics

**Fig. 4:**
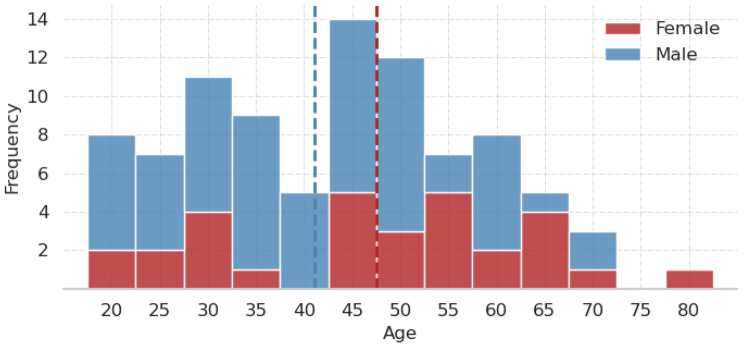
Stacked age histogram of the study cohort (90 out of 91 subjects), grouped by sex. The age of one subject was not specified (missing value). The dashed vertical lines denote the means of the respective groups. The mean age of the entire cohort is approximately 43 years. The age range spans from 18 to 82 years

The dataset comprised 200 MRI scans from 91 unique individuals, with a predominance of male participants (60 males, 30 females, and one undefined). The age distribution, stratified by sex, is illustrated in Fig. 4. More than half of the participants contributed multiple scans, with some individuals providing up to seven. Each original–defaced scan pair was treated as an independent data point, as the proposed method assesses defacing quality at the image level rather than relying on subject-specific features. However, to prevent potential data leakage arising from multiple scans per participant, we implemented a nested stratified group k-fold cross-validation framework, in which the group component ensured that all scans from a single individual were assigned to the same fold, thereby enforcing strict subject-level separation [33]. This dataset provides an initial foundation for evaluating defacing methods and benchmarking automated ML models in defacing QA.

### Defacing performance

**Fig. 5:**
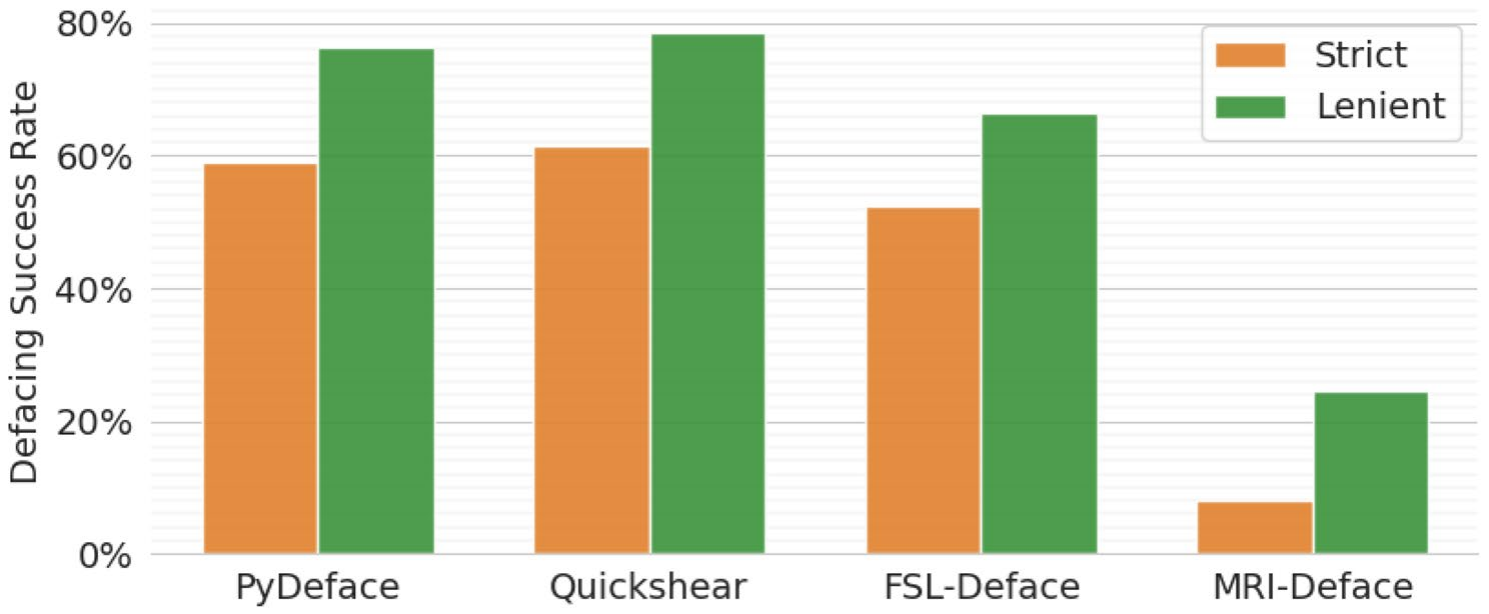
Success rates of adequately defaced MRI scans, manually evaluated under both strict and lenient defacing criteria. The overall success rates were higher under lenient criteria. However, MRI-Deface consistently exhibited the lowest performance, with its success likely underestimated due to residual facial features

The results of the manual defacing assessments are shown in Fig. 5. Among the evaluated defacing methods, PyDeface exhibited the highest success rate, followed closely by Quickshear and FSL-Deface. In contrast, MRI-Deface demonstrated the lowest performance, with only approximately 5% of scans meeting the strict defacing criterion and about 20% identified as adequately defaced under the lenient criterion. Consequently, the majority of failed defaced scans were attributed to the MRI-Deface.

Unexpectedly, all defacing algorithms occasionally introduced minor voxel intensity alterations across nearly the entire MRI scan. This effect was particularly pronounced in MRI-Deface and was observed across different MRI modalities, including T1 and FLAIR. Furthermore, inconsistencies in BHRs were noted, with certain brain masks occupying either an excessive or insufficient proportion of the head volume, indicating variability in mask accuracy.

### Feature correlation and selection

**Fig. 6:**
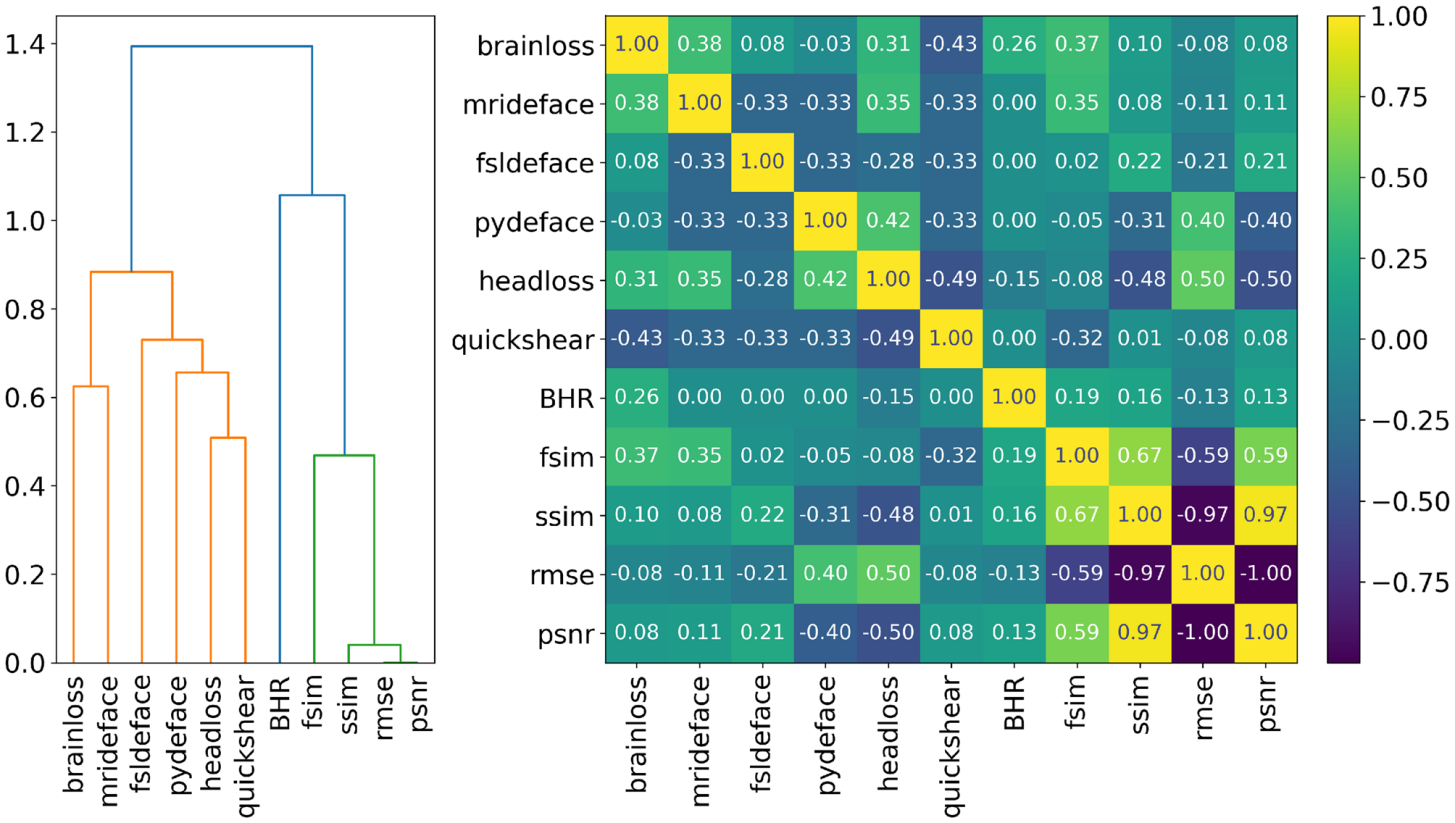
Hierarchical clustering dendrogram and correlation heatmap based on Spearman’s rank-order correlation. BHR represents the ratio of brain tissue to total head tissue voxels, indicating potential variations in brain mask quality. The “brain loss” and “head loss” refer to the percentage of removed voxels from the brain mask and total head volume, respectively

To improve ML model performance and minimize feature redundancy, a correlation analysis was conducted. The resulting heatmap, shown in Fig. 6, illuminates the relationships among the extracted features. Notably, RMSE, PSNR, and SSIM exhibited strong intercorrelations, suggesting potential redundancy in the feature set. The strong correlation between RMSE and PSNR is expected, given that PSNR is directly derived from RMSE. In contrast, FSIM showed only moderate correlation with these metrics, setting it apart from other similarity measures. Additionally, FSIM demonstrated a moderate correlation with the defacing success, indicating its potential relevance for classification. These observations informed subsequent feature selection addressed in Sect. 2.

To refine the ML feature set, permutation feature importance was computed and combined with the Spearman rank-order hierarchical clustering dendrogram to guide feature selection. The initial set comprised 11 features, including four image similarity metrics, three volume-based measures, and four defacing methods introduced in Sect. 2. Across all experimental conditions, FSIM consistently emerged as the most influential feature. In the absence of defacing methods as predictors, tree-based models (RF and XGB) showed increased reliance on image-based metrics, particularly the percentage of removed brain and head voxels, with FSIM, head loss, and brain loss consistently ranking as top contributors. When defacing methods were included, MRI-Deface exhibited high importance, likely reflecting its comparatively poorer defacing performance. LR showed greater variability in feature importance, yet it generally emphasized FSIM, brain loss, and either RMSE or SSIM, depending on the feature set. The MLP model demonstrated a broader range of features, but generally assigned lower importance to PSNR and BHR. Given the strong and stable performance of RF and XGB, the feature set was reduced to eight key predictors: FSIM, SSIM, the percentage of removed head and brain tissue, and the four defacing methods. Although MRI-Deface was the most influential single feature, all four defacing tools were retained due to their collective contribution to model performance, each providing complementary discriminatory information that enhanced overall classification accuracy.

### ML classifier performance

The performance of the ML models, as summarized in Table 1, demonstrates their efficacy in distinguishing adequately defaced MRI scans from those with residual facial features. RF and XGB achieve the highest classification performance under the strict defacing criterion, with MLP performing slightly lower. In contrast, LR struggles to balance sensitivity and specificity, yielding suboptimal results compared to the other models.

**Table 1:** Performance of ML models in classifying defacing success, including defacing method labels, evaluated under strict and lenient defacing criteria. Features included FSIM, SSIM, percentage of removed brain and head tissue, and four defacing methods. Reported values are averaged over nested cross-validation folds, with standard deviations shown in parentheses

	Model	Accuracy	Specificity	Sensitivity	Precision	AUROC
Strict	LR	0.80 (0.07)	0.90 (0.06)	0.73 (0.12)	0.89 (0.06)	0.82 (0.05)
	MLP	0.79 (0.06)	0.85 (0.11)	0.75 (0.12)	0.87 (0.08)	0.80 (0.05)
	RF	0.81 (0.07)	0.89 (0.06)	0.76 (0.11)	0.89 (0.05)	0.82 (0.05)
	XGB	0.80 (0.07)	0.85 (0.08)	0.77 (0.10)	0.86 (0.07)	0.81 (0.05)
Lenient	LR	0.84 (0.06)	0.86 (0.08)	0.80 (0.06)	0.79 (0.11)	0.83 (0.05)
	MLP	0.84 (0.04)	0.89 (0.06)	0.78 (0.07)	0.81 (0.09)	0.83 (0.04)
	RF	0.85 (0.04)	0.86 (0.05)	0.82 (0.08)	0.79 (0.08)	0.84 (0.04)
	XGB	0.84 (0.04)	0.86 (0.06)	0.81 (0.08)	0.78 (0.09)	0.83 (0.04)

Under the lenient defacing criterion, RF and XGB continue to outperform the other models, exhibiting notably higher sensitivity compared to LR and MLP. These findings indicate that as the classification criteria become more permissive, those models improve their ability to differentiate adequately defaced from inadequately ones. This trend suggests that tree-based ensemble methods may be more adept at capturing subtle variations in defacing quality, potentially due to their ability to model complex, non-linear relationships in the feature space. Future research should further explore the robustness of these models under varying defacing thresholds and assess their generalizability across diverse datasets.

## Discussion

The assessment of defacing success in MRI scans has been relying on manual or semi-automated methods, underscoring the need for more efficient and scalable approaches [7, 12, 13, 34]. Delbarre et al. addressed this gap by implementing a convolutional neural network (CNN) for automated QA of MRIs defaced using FSL-Deface [17]. While CNNs demonstrated promising results, their reliance on large labeled datasets limits their adaptability to new defacing algorithms without extensive manual annotations. In contrast, the present study introduces DefaceQA, a data-efficient ML approach that evaluates defacing quality using quantitative image features. By extracting interpretable similarity metrics between defaced and original scans, DefaceQA enables robust classification of defacing adequacy. This approach mitigates challenges observed in previous studies, including high inter-rater variability and subjectivity in defining defacing success.

### Impact of manual rating

Inter-rater variability remains a major challenge in manual defacing evaluation, particularly in cases where distinguishing facial features is difficult [12]. Theyers et al. initially applied a strict defacing criterion that required the complete removal of facial features but later adopted a more lenient approach due to challenges in recognizing facial structures from rendered images. In contrast, DefaceQA utilized side-by-side comparisons of original and defaced MRIs from multiple perspectives, providing raters with additional contextual information that may have improved rating consistency.

However, discrepancies between our findings and those of Theyers et al. highlight the impact of classification strictness on defacing success rates. For instance, the present study identified an 75% error rate for MRI-Deface, compared to 15% reported by Theyers et al., likely due to the preservation of recognizable eye structures post-defacing. Similarly, while Theyers et al. reported a 60% error rate for QuickShear, our assessment found only 20%, potentially due to differences in how residual internal eye structures were classified. In the present study, these structures were deemed non-identifiable and therefore not compromising privacy, underscoring rating methodology influence defacing assessments.

The integration of original-reference comparisons in our evaluation facilitated a more detailed analysis, resulting in a higher proportion of scans being classified as insufficiently defaced. These findings emphasize the complexity of defacing assessment and highlight the necessity for standardized evaluation criteria to enhance both consistency and privacy protection in neuroimaging research.

To assess the reliability of manual defacing evaluations, three raters classified each scan independently using strict and lenient criteria. Table 2 summarizes the inter-rater agreement for each defacing method. The extent of agreement was measured using both raw percent agreement and Cohen’s kappa coefficient (*κ*), which accounts for chance-level agreement. Across methods, there was substantial agreement under the lenient criteria, with PyDeface and FSL-Deface achieving particularly strong reliability (*κ* = 0.90 and *κ* = 0.88, respectively). The MRI-Deface model demonstrated the highest degree of agreement under the strict criterion (95.90%), but showed lower *κ* values, likely due to class imbalance.

**Table 2:** Inter-rater reliability for manual evaluation of defacing success across four defacing methods. Agreement is reported for both strict and lenient evaluation criteria using percent agreement and Cohen’s kappa (*κ*).

Defacing method	Strict		Lenient	
	Agreement (%)	***κ***	Agreement (%)	***κ***
PyDeface	75.72	0.52	95.47	0.90
Quickshear	76.13	0.55	91.36	0.82
FSL-Deface	85.60	0.71	93.83	0.88
MRI-Deface	95.90	0.65	90.98	0.69

### Quantitative features and similarity metrics

ML models, trained on extracted quantitative similarity metrics, demonstrated a high degree of robustness, with FSIM emerging as the most influential feature across all conditions. FSIM, designed for image QA by emphasizing low-level salient features, exhibits a strong alignment with human visual perception [35]. This characteristic positions FSIM as an essential component contributing to the predictive capability of the ML models. The congruence between FSIM and human perception is thought to contribute to the model’s superior performance.

Notably, despite utilizing a smaller subset of features, DefaceQA achieved performance only slightly lower than that of the CNN developed by Delbarre et al. [17]. We note that the reported 92% accuracy in their study was obtained by applying a strict probability threshold, which resulted in only 45% of the data being classified with high confidence. Moreover, their model was limited to the FSL-Deface method and used four outcome categories; when all failures types were grouped together, the overall accuracy was 82%. This emphasizes the efficiency of the present approach, which does not require large labeled datasets and demonstrates flexibility across various defacing techniques. The results underscore the sufficiency of quantitative metrics, such as FSIM, for achieving robust model performance, thereby supporting the validity and potential of this methodology in defacing QA.

The model performance, as detailed in Sect. 3, was evaluated using a feature set that included labels corresponding to defacing methods. To enhance generalizability and develop a method-independent approach, four features associated with specific defacing algorithms were excluded. The updated results, shown in Table 3, indicate that the model retains strong efficacy, albeit with a marginal decline in performance. Notably, under the strict criterion, specificity is more affected than sensitivity, suggesting an increased susceptibility to false positives when the model lacks information about the applied defacing technique.

**Table 3:** Performance of ML models in classifying defacing success, excluding defacing method labels, evaluated under strict and lenient defacing criteria. Features included FSIM, SSIM, and the percentage of removed brain and head tissue. Reported values are averaged over nested cross-validation folds, with standard deviations shown in parentheses

	Model	Accuracy	Specificity	Sensitivity	Precision	AUROC
Strict	LR	0.77 (0.06)	0.81 (0.08)	0.74 (0.10)	0.82 (0.08)	0.78 (0.06)
	MLP	0.76 (0.07)	0.75 (0.13)	0.78 (0.11)	0.80 (0.10)	0.77 (0.07)
	RF	0.78 (0.06)	0.81 (0.09)	0.74 (0.09)	0.83 (0.08)	0.78 (0.06)
	XGB	0.77 (0.07)	0.81 (0.10)	0.75 (0.09)	0.83 (0.09)	0.78 (0.06)
Lenient	LR	0.81 (0.06)	0.84 (0.07)	0.77 (0.11)	0.75 (0.11)	0.81 (0.07)
	MLP	0.80 (0.07)	0.88 (0.06)	0.66 (0.16)	0.77 (0.11)	0.77 (0.09)
	RF	0.82 (0.05)	0.86 (0.06)	0.74 (0.12)	0.77 (0.10)	0.80 (0.06)
	XGB	0.81 (0.07)	0.82 (0.06)	0.78 (0.13)	0.73 (0.10)	0.80 (0.07)

### Limitations and future directions

Despite the promising findings of this study, several limitations must be acknowledged. First, the dataset consisted of only 91 distinct individuals, which raises concerns regarding the potential impact of facial similarities across multiple scans on the model’s generalizability. To address this, future research should aim to expand the dataset, incorporating more diverse samples such as those from the publicly available IXI MRI dataset [36], to improve the robustness and generalizability of the model.

Defacing efficacy was manually assessed by three independent raters. Two primary raters evaluated all scans, while a third rater adjudicated only the discrepant cases. Final labels were determined by majority vote, with any remaining disagreements resolved through consensus. This multi-rater strategy aimed to improve the reliability and consistency of the ground truth annotations. However, some degree of subjectivity and inter-rater variability persists, particularly in borderline cases where the success of defacing was ambiguous, which should be noted as a limitation.

In addition, the brain extraction algorithm used in the present study may have introduced inaccuracies in the BHR calculations, particularly if brain masks were inconsistently generated. This could lead to unreliable estimates of brain tissue removal. Therefore, further refinement of the brain extraction pipeline is needed to ensure more accurate preprocessing. Although image quality was not explicitly modeled in this study, it is likely to influence both defacing outcomes and the performance of automated QA models. As the quality of input data affects the performance of ML models, optimizing preprocessing steps, such as improving brain mask accuracy, will be critical for enhancing the model reliability. Future work could also investigate the incorporation of objective image quality metrics as additional predictive features.

While demographic and scanner-related variables such as sex, age, magnetic field strength, and scanner type were not included as predictors in this study, their potential impact on defacing performance warrants further investigation. Future work should systematically examine these factors, as they may contribute to variability in outcomes and could enhance the predictive power of defacing QA models. Additionally, due to the highly imbalanced distribution of MRI sequence types in our dataset, predominantly T1-weighted and FLAIR images with only a single T2-weighted scan, we excluded the latter from all analyses to avoid introducing bias. However, MRI sequence modality may influence defacing success, as different modalities (e.g., T1-weighted vs. FLAIR) can vary in structural contrast and visibility of facial features. A more balanced dataset including diverse MRI types will be essential for evaluating the predictive value of sequence modality in future studies.

Moreover, while this study adopts a supervised learning approach using manually labeled data to ensure rigorous and interpretable defacing QAs, future research could benefit from incorporating unsupervised or self-supervised DL methods. These approaches may be particularly advantageous for large-scale datasets, enabling the identification of defacing failure patterns without requiring extensive manual annotation. Additionally, the integration of radiomics-based features, using tools such as PyRadiomics, offers another promising direction [37]. These features can capture nuanced textural and spatial patterns associated with defacing outcomes and may further enhance model performance, especially when applied to more diverse and expansive image datasets.

Lastly, the risk of false-negative misclassifications remains a significant concern, particularly in the context of neuroimaging anonymization where privacy is of paramount. To address this issue, future iterations of the model should incorporate stricter decision thresholds, in line with the approach taken by Delbarre et al., to minimize privacy risks. Despite these limitations, the present study represents an important step toward automating defacing QA, with the potential to reduce the manual workload in large-scale neuroimaging anonymization workflows.

## Conclusions

This study advances the automated evaluation of defacing algorithms by leveraging conventional ML models and quantitative similarity metrics employed by DefaceQA. This approach presents a data-efficient alternative to CNN-based methods, offering a scalable and generalizable solution that minimizes manual labeling efforts while enhancing privacy protection in neuroimaging research.

Integrating DefaceQA into anonymization pipelines has the potential to improve compliance and security in data-sharing practices, promoting responsible and ethical research. Future work will focus on refining the methodology, expanding dataset diversity, and enhancing model robustness, to facilitate broader adoption of this approach in defacing QA workflows.

## Data Availability

The datasets used or analyzed during the current study are not publicly available due to patient privacy concerns but are available from the corresponding author on reasonable request. The data obtained from the Leukodystrophy Registry of the Clinic and Polyclinic for Neurology at the Leipzig University Medical Center.

## Code availability

The underlying code for this study is available in the GitHub repository and can be accessed via this link https://github.com/ul-mds/defacing-evaluation.
